# Strontium Promotes the Proliferation and Osteogenic Differentiation of Human Placental Decidual Basalis- and Bone Marrow-Derived MSCs in a Dose-Dependent Manner

**DOI:** 10.1155/2019/4242178

**Published:** 2019-11-22

**Authors:** Yi-Zhou Huang, Cheng-Guang Wu, Hui-Qi Xie, Zhao-Yang Li, Antonietta Silini, Ornella Parolini, Yi Wu, Li Deng, Yong-Can Huang

**Affiliations:** ^1^Laboratory of Stem Cell and Tissue Engineering, State Key Laboratory of Biotherapy and Cancer Center, West China Hospital, Sichuan University and Collaborative Innovation Center of Biotherapy, Chengdu, Sichuan 610041, China; ^2^Department of Orthopaedics, West China Hospital, Sichuan University, Chengdu, Sichuan 610041, China; ^3^School of Materials Science and Engineering, Tianjin University, Tianjin 300072, China; ^4^Centro di Ricerca E. Menni, Fondazione Poliambulanza-Istituto Ospedaliero, Brescia 25124, Italy; ^5^Istituto di Anatomia Umana e Biologia Cellulare, Università Cattolica del Sacro Cuore Facoltà di Medicina e Chirurgia, Roma 00168, Italy; ^6^Medical College, Jinggangshan University, Ji'an, Jiangxi 343009, China; ^7^Shenzhen Engineering Laboratory of Orthopaedic Regenerative Technologies, Orthopaedic Research Center, Peking University Shenzhen Hospital, Shenzhen, Guangdong 518036, China; ^8^Shenzhen Key Laboratory of Spine Surgery, Department of Spine Surgery, Peking University Shenzhen Hospital, Shenzhen, Guangdong 518036, China; ^9^National & Local Joint Engineering Research Center of Orthopaedic Biomaterials, Peking University Shenzhen Hospital, Shenzhen, Guangdong 518036, China

## Abstract

The osteogenic potential of mesenchymal stromal cells (MSCs) varies among different tissue sources. Strontium enhances the osteogenic differentiation of bone marrow-derived MSCs (BM-MSCs), but whether it exerts similar effects on placental decidual basalis-derived MSCs (PDB-MSCs) remains unknown. Here, we compared the influence of strontium on the proliferation and osteogenic differentiation of human PDB- and BM-MSCs *in vitro*. We found that 1 mM and 10 mM strontium, but not 0.1 mM strontium, evidently promoted the proliferation of human PDB- and BM-MSCs. These doses of strontium showed a comparable alkaline phosphatase activity in both cell types, but their osteogenic gene expressions were promoted in a dose-dependent manner. Strontium at doses of 0.1 mM and 1 mM elevated several osteogenic gene expressions of PDB-MSCs, but not those of BM-MSCs at an early stage. Nevertheless, they failed to enhance the mineralization of either cell type. By contrast, 10 mM strontium facilitated the osteogenic gene expression as well as the mineralization of human PDB- and BM-MSCs. Collectively, this study demonstrated that human PDB- and BM-MSCs shared a great similarity in response to strontium, which promoted their proliferation and osteogenic differentiation in a dose-dependent manner.

## 1. Introduction

Large bone defects, resulting from trauma, infection, and congenital diseases, remain a great challenge in the clinic. Mesenchymal stromal cells (MSCs) are fibroblast-like adherent cells with a self-renewal ability and multipotency. They are considered as promising seed cells for bone regeneration, mainly due to their osteogenic potential and paracrine effects [1, 2]. Currently, bone marrow represents the main source of MSCs for clinical studies. A great number of animal studies and several clinical trials have shown that bone marrow-derived MSCs (BM-MSCs) improved bone regeneration [2, 3]. However, several drawbacks hinder wide clinical application. For example, the isolation of BM-MSCs requires an invasive procedure, and the proliferation and differentiation capability of BM-MSCs decrease with donor age [4, 5]. Therefore, many researches tried to explore other sources of MSCs for bone regeneration.

The human placenta is an attractive source of MSCs because of the noninvasive tissue collection, the lack of ethical concerns, the high cell harvest rate, and the robust cell proliferation ability [6]. MSCs have been isolated from different parts of the placenta, including the chorionic villi, amnion membrane, and decidual basalis [7–10]. They can undergo osteogenic differentiation when cultured in the traditional osteogenic medium or stimulated by the osteogenic growth factors (e.g., bone morphogenic proteins) *in vitro* [11]. When grafted in combination with osteoinductive scaffolds, they are able to form bone tissue at the ectopic sites [12, 13]. Noticeably, they also enhanced bone regeneration after transplantation at the bone defects [14, 15]. Nevertheless, several studies have revealed that the osteogenic potential of placenta-derived MSCs was inferior to that of the BM-MSCs [16–18], highlighting the need of developing efficient strategies to improve the osteogenic commitment of cells.

Strontium is a trace element in natural bone tissue that has dual effects on bone metabolism. It enhances the proliferation of osteoprogenitor cells, while inhibiting the terminal differentiation of osteoclasts [19]. Considering the chemical stability and low cost of strontium, many efforts have been made in developing strontium-modified scaffolds to enhance bone repair in combination with MSCs [20, 21]. It is well-known that strontium promotes the osteogenic differentiation of osteoblast and BM-MSCs derived from rodent species [22–28]. However, much fewer studies have determined the effect of strontium on MSCs derived from human tissues [29–32], especially those derived from perinatal tissues. Yang et al. has reported the enhanced osteogenic response of human umbilical cord-derived MSCs to strontium stimulation [32], but the influence of strontium on MSCs derived from other human perinatal tissues, such as the decidual basalis, has not been investigated yet.

Since tissue origin profoundly influences the biological properties of MSCs, including their proliferation ability and differentiation potential [16–18, 33], it is reasonable to assume that human BM-MSCs and placental decidual basalis-derived MSCs (PDB-MSCs) might respond differently to strontium stimulation. In this study, we first determined the proper treatment doses of strontium for both cell types and then compared the effect of strontium on their osteogenic differentiation *in vitro*, with the aim of providing valuable information for potential applications of strontium for MSC-based bone regeneration.

## 2. Materials and Methods

### 2.1. Cell Isolation

This study was approved by the ethics committee of West China Hospital, Sichuan University. Human placentas were obtained from four healthy donors (age ranging from 25 to 33) with informed consent. PDB-MSCs were isolated as described in our previous report [7]. Briefly, decidua basalis was dissected from placentas, washed in phosphate-buffered saline (PBS), minced into small pieces of tissue, and digested with 0.25% Trypsin (Gibco, USA) and 0.1% Collagenase IV (Invitrogen, USA). After centrifugation, the nucleated cells were resuspended and cultured in the growth medium containing Dulbecco's modified Eagle's medium-high glucose (Gibco, USA), 10% fetal bovine serum (HyClone, USA), and 1% penicillin/streptomycin (Gibco, USA). The growth medium was changed every 3 days. When the cells reached 70-80% confluence, they were collected by 0.25% trypsin/ethylene diamine tetraacetic acid (EDTA; Sigma-Aldrich, USA) treatment and passaged at a dilution of 1 : 3. Cells from the 4th passage of each donor were pooled together, and the mixed cells were cultured for an additional 1 to 3 passages for further use in the subsequent studies.

Human bone marrow samples were obtained from one healthy female donor (25 years old) and three patients with scoliosis (one female donor and two male donors, age ranging from 15 to 18) with informed consent. BM-MSCs were isolated according to our previous description [34]. Briefly, bone marrow aspirates were diluted with PBS, layered over Ficoll solution (TBD Science, China), and centrifuged at 500 g for 30 mins to collect mononuclear cells from the gradient interface. Then, the mononuclear cells were cultured in the growth medium, which was changed to remove the nonadherent cells after 72 hours of culture. When the cells reached 70-80% confluence, they were passaged at a ratio of 1 : 3. The cells at passage 4 from each donor were pooled together, and the mixed cells were subcultured for additional passages. Cells at the 5th to 7th passage were used in the following experiments.

### 2.2. Cell Characterization

The multilineage differentiation potential of human BM-MSCs and PDB-MSCs was investigated according to our previous reports [7, 34]. Briefly, the cells were seeded at a density of 1 × 10^4^ cells/cm^2^. For osteogenic differentiation, the cells were cultured in the osteogenic medium containing the growth medium, 50 mg/L L-ascorbic acid-2-phosphate (Sigma-Aldrich, USA), 10^−7^ M dexamethasone (Sigma-Aldrich, USA), and 10 mM *β*-glycerophosphate (Sigma-Aldrich, USA). The osteogenic medium was changed every 3 days. After induction for 21 days, the samples were fixed in 75% ethanol and stained with 1% alizarin red solution (Sigma-Aldrich, USA) for 30 mins at 37°C.

For adipogenic differentiation, the cells were cultured in the adipogenic medium containing the growth medium, 0.25 *μ*M dexamethasone (Sigma-Aldrich, USA), 10 *μ*M insulin (Sigma-Aldrich, USA), 0.5 *μ*M isobutyl-methylxanthine (Sigma-Aldrich, USA), and 50 *μ*M indomethacin (Sigma-Aldrich, USA). The adipogenic medium was changed every 3 days. After induction for 15 days, the samples were fixed with 10% formalin and stained with oil red O solution (Sigma-Aldrich, USA) for 45 mins to detect lipid droplets in the cytoplasm.

For chondrogenic differentiation, the cells were cultured in the chondrogenic medium containing the growth medium, 100 mg/L sodium pyruvate (Sigma-Aldrich, USA), 10 *μ*g/L transforming growth factor-*β*1 (R&D Systems, USA), 100 mg/L ascorbate-2-phosphate (Sigma-Aldrich, USA), 1% insulin-transferrin-selenium (Gibco, USA), and 0.1 *μ*M dexamethasone (Sigma-Aldrich, USA). The chondrogenic medium was changed every 3 days. After induction for 14 days, the samples were stained with Alcian Blue 8GX (Cyagen Biosciences, China) for 30 mins to observe the deposition of glycosaminoglycans. After histological staining, all of the samples were observed and photographed by a phase-contrast microscope (Olympus Corporation, Japan).

### 2.3. Cell Proliferation

Both BM- and PDB-MSCs were cultured in the growth medium or the growth medium supplemented with different concentrations of SrCl_2_·6H_2_O (Kelong, China), respectively. The cells cultured in the growth medium served as the control group. Briefly, the cells were seeded at a density of 3000 cells/well in 96-well plates. Cell proliferation was continually monitored using an alamarBlue® Cell Viability Reagent (Invitrogen, USA) on days 1, 3, and 7. At each timepoint, the cells were rinsed with PBS and then incubated with 100 *μ*L working solution prepared according to the manufacturer's instructions for 4 h at 37°C. Finally, the absorbance was recorded using a plate reader (Molecular Devices, USA) at 570 nm, using 600 nm as a reference wavelength.

### 2.4. Live/Dead Staining

Human BM-MSCs were seeded at a density of 5,000 cells/cm^2^ in 12-well plates and cultured in the growth medium or the growth medium supplemented with strontium at doses of 0.1 mM, 1 mM, and 10 mM, respectively. On days 1, 3, and 7, a Live/Dead® Cell Viability Assay Kit (Invitrogen, USA) was used to monitor cell viability. The samples (*n* = 3 for each group) were gently washed with PBS for two times and then incubated in the working solution for 30 mins at 37°C. Cell viability was observed by an inverted fluorescence microscope (Olympus Corporation, Japan).

### 2.5. Cell Apoptosis Analysis

Human BM-MSCs were seeded at a density of 5,000 cells/cm^2^ in 25 cm^2^ culture flasks and cultured in the growth medium or the growth medium supplemented with different doses of strontium (0.1 mM, 1 mM, and 10 mM) for 3 days, respectively. The cells cultured in the growth medium served as the control group. After cell culture, the cells were harvested for apoptosis detection (Annexin V-FITC Apoptosis Detection Kit, Beyotime Institute of Biotechnology, China) according to the manufacturer's instructions, and the samples were analysed using flow cytometry (FACSAria II, BD Biosciences, USA).

### 2.6. Alkaline Phosphatase (ALP) Activity Assay

Human BM- and PDB-MSCs were seeded at a density of 5000 cells/cm^2^ and cultured in the growth medium or the osteogenic medium added with different doses of strontium (0.1 mM, 1 mM, and 10 mM), respectively. The cells cultured in the growth medium or the osteogenic medium served as the control group, respectively. After 7 days of culture, the ALP activity of cells (*n* = 4 for each group) was measured using an ALP assay kit (Nanjin Jiancheng Bioengineering Institute, China). Briefly, the cells were detached by 0.25% trypsin/EDTA, resuspended in deionized H_2_O (100 *μ*L/sample), and lysed by three freeze-thaw cycles. Then, the ALP activity of cell lysates was determined according to the manufacturer's instructions. The absorbance at 405 nm was read by a plate reader (Molecular Devices, USA). The ALP activity results were normalized to the amount of total protein, which was quantified using a BCA protein assay kit (Bio-Rad, USA).

### 2.7. ALP Staining

Human BM- and PDB-MSCs were seeded at a density of 5000 cells/cm^2^ and cultured with different doses of strontium (0.1 mM, 1 mM, and 10 mM) supplemented in the growth medium or the osteogenic medium, respectively. The cells cultured in the growth medium or the osteogenic medium served as the control group, respectively. After 7 days of culture, the ALP activity of cells (*n* = 3 for each group) was observed using an ALP staining kit (Sigma-Aldrich, USA). Briefly, the cells were fixed in 4% phosphate-buffered paraformaldehyde for 30 mins at room temperature, washed with running water, stained with an ALP staining kit according to the manufacturer's instructions, and finally observed using microscopy (Olympus Corporation, Japan).

### 2.8. Real-Time Polymerase Chain Reaction (RT-PCR)

Human BM- and PDB-MSCs were seeded at a density of 5000 cells/cm^2^ and cultured with different doses of SrCl_2_·6H_2_O (0.1 mM, 1 mM, and 10 mM) supplemented in the growth medium or the osteogenic medium, respectively. The cells cultured in the growth medium or the osteogenic medium served as the control group, respectively. After culture for 3, 7, and 14 days, total RNA (*n* = 4 for each group) was extracted using a RNAiso Plus reagent (Takara Bio, Japan) and then reverse-transcribed into cDNA using a PrimeScript RT Reagent Kit (Takara Bio, Japan). Gene expression was quantified using a SYBR Premix Ex Taq II Kit (Takara Bio, Japan) in an iQ5 real-time system (Bio-Rad, USA). The primers for the osteogenic genes and the housekeeping gene, including *runt-related transcription factor 2* (*Runx2*), *osteocalcin* (*OC*), *osteoprotegerin* (*OPG*), *osteopontin* (*OPN*), and *glyceraldehyde-3-phosphate dehydrogenase* (*GADPH*), are listed as follows: *Runx 2*: forward primer—CCCAGTATGAGAGTAGGTGTCC, reverse primer—GGGTAAGACTGGTCATAGGACC; *OC*: forward primer—GAGGGCAGCGAGGTAGTGAA, reverse primer—TCCTGAAAGCCGATGTGGTC; *OPN*: forward primer—TGACCAGAGTGCTGAAACCCA, reverse primer—CCTGACTATCAATCACATCGGAAT; *OPG*: forward primer—GGTCTCCTGCTAACTCAGAAAGG, reverse primer—CAGCAAACCTGAAGAATGCCTCC; and *GAPDH*: forward primer—CTTTGGTATCGTGGAAGGACTC, reverse primer—GTAGAGGCAGGGATGATGTTCT. *GADPH* served as the housekeeping gene. Target gene expression was analyzed by the 2^-△△Ct^ method. Results were expressed relative to the gene expression level of the control group.

### 2.9. Mineralization Assay

Human BM- and PDB-MSCs were seeded at a density of 5000 cells/cm^2^ in 6-well plates and were cultured in the osteogenic medium supplemented with different doses of strontium (0.1 mM, 1 mM, and 10 mM) for 21 days, respectively. The cells cultured in the osteogenic medium served as the control group. After induction, the samples (*n* = 3 for each group) were stained with alizarin red as described above and were grossly visualized.

### 2.10. Statistical Analysis

Data were expressed as the mean ± standard deviation (SD). A one-way ANOVA followed by Dennett's Multiple Comparison Test was performed for statistical testing using GraphPad Prism software (GraphPad Software Inc., USA), and *p* < 0.05 was considered significant.

## 3. Results

### 3.1. Strontium Enhanced the Proliferation of BM- and PDB-MSCs

**Figure 1:**
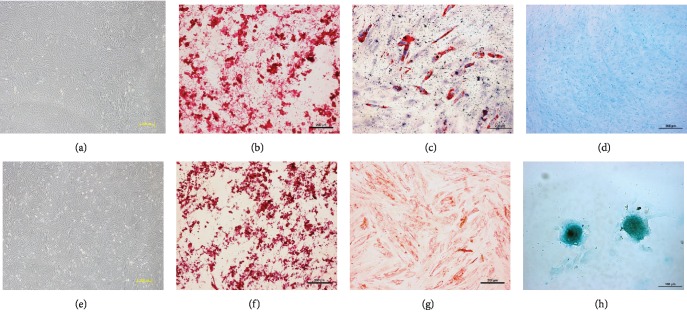
Characterization of human BM- and PDB-MSCs. Morphology of (a) BM-MSCs and (e) PDB-MSCs; scale bar: 200 *μ*m. Alizarin red staining of (b) BM-MSCs and (f) PDB-MSCs after osteogenic induction; scale bar: 200 *μ*m. Oil red O staining of (c) BM-MSCs and (g) PDB-MSCs after adipogenic induction; scale bar: 200 *μ*m. Alcian blue staining of (d) BM-MSCs and (h) PDB-MSCs after chondrogenic induction; scale bar: 200 *μ*m for (d) and 100 *μ*m for (h).

Human BM- and PDB-MSCs were characterized before investigating their responses to strontium stimulation. Similar to our previous reports [7, 34], both cells showed a fibroblastic-like morphology and possessed the osteogenic, adipogenic, and chondrogenic differentiation potential *in vitro* (Figure 1). Interestingly, after chondrogenic induction, both BM- and PDB-MSCs were positive for Alcian blue staining, but there was an obvious difference between them: a multilayered cell sheet was observed in BM-MSCs, while PDB-MSCs formed cellular nodules. This result indicates a tissue origin-dependent variation in the chondrogenic potential of MSCs, which has been reported by other research groups [35].

**Figure 2:**
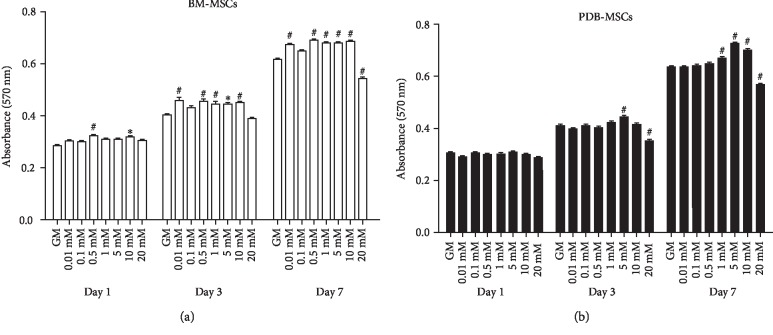
Proliferation of BM- and PDB-MSCs cultured with different doses of strontium supplemented in the growth medium. (a) BM-MSCs. (b) PDB-MSCs. ^∗^*P* < 0.05 when compared with the GM group; ^#^*P* < 0.01 when compared with the GM group. GM: growth medium. All of these results are representative of three independent experiments.

To determine the proper doses of strontium, both cell types were cultured with different concentrations of strontium (0.01 mM, 0.1 mM, 0.5 mM, 1 mM, 5 mM, 10 mM, and 20 mM). The cell proliferation was evaluated on days 1, 3, and 7. As shown in Figure 2(a), strontium, at doses of 0.01 mM, 0.5 mM, 1 mM, 5 mM, and 10 mM, facilitated the growth of BM-MSCs, while 20 mM strontium inhibited the replication. For PDB-MSCs, the growth rate of cells treated with 0.01 mM, 0.1 mM, and 0.5 mM strontium was comparable to the GM group at all timepoints. 1 mM, 5 mM, and 10 mM strontium promoted the proliferation of PDB-MSCs on day 7, while 20 mM strontium showed an evident inhibition on days 3 and 7 (Figure 2(b)).

**Figure 3:**
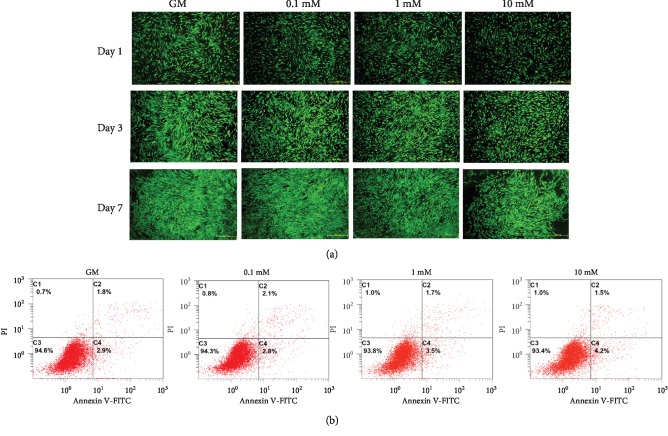
Cytotoxic effects of strontium on human BM-MSCs. (a) Live/dead staining of human BM-MSCs cultured with different concentrations of strontium; green fluorescence: live cells; red fluorescence: dead cells; scale bar: 500 *μ*m. (b) Cell apoptosis analysis of human BM-MSCs. The percentages of the following cell populations are indicated on the respective density plots: necrotic cells (Annexin V-negative and PI-positive) are located in the upper left (C1), late apoptotic cells (Annexin V-positive and PI-positive) in the upper right (C2), viable cells (Annexin V-negative and PI-negative) in the lower left (C3), and early apoptotic cells (Annexin V-positive and PI-negative) in the lower right (C4) quadrants, respectively. GM: growth medium. All of these results are representative of three independent experiments.

To further confirm the cytotoxic effects of strontium on human BM-MSCs, live/dead staining and cell apoptosis analysis were performed. As shown in Figure 3(a), the cells showed good viability (green fluorescence) on days 1, 3, and 7, and few dead cells (red fluorescence) were observed in each group. Similarly, the result of an apoptosis assay revealed a comparable cell viability in the 0.1 mM, 1 mM, and 10 mM strontium groups when comparing with the GM group (Figure 3(b)).

Considering the fact that the average strontium concentration in the serum of postmenopausal women taking 2 g/day strontium ranelate orally is 0.117 mM [36], and combining this information with the above results, we therefore chose 0.1 mM, 1 mM, and 10 mM strontium in the subsequent experiments to investigate the osteogenic effects of strontium on BM- and PDB-MSCs.

### 3.2. Strontium Promoted the Osteogenic Differentiation of BM- and PDB-MSCs in the Growth Medium

To determine whether strontium promoted the osteogenic differentiation of human BM- and PDB-MSCs without any other soluble osteogenic factors, both cells were cultured in the growth medium supplemented with different doses of strontium. Then, the osteogenic differentiation of cells was measured, including the ALP activity and the osteogenic gene expression.

**Figure 4:**
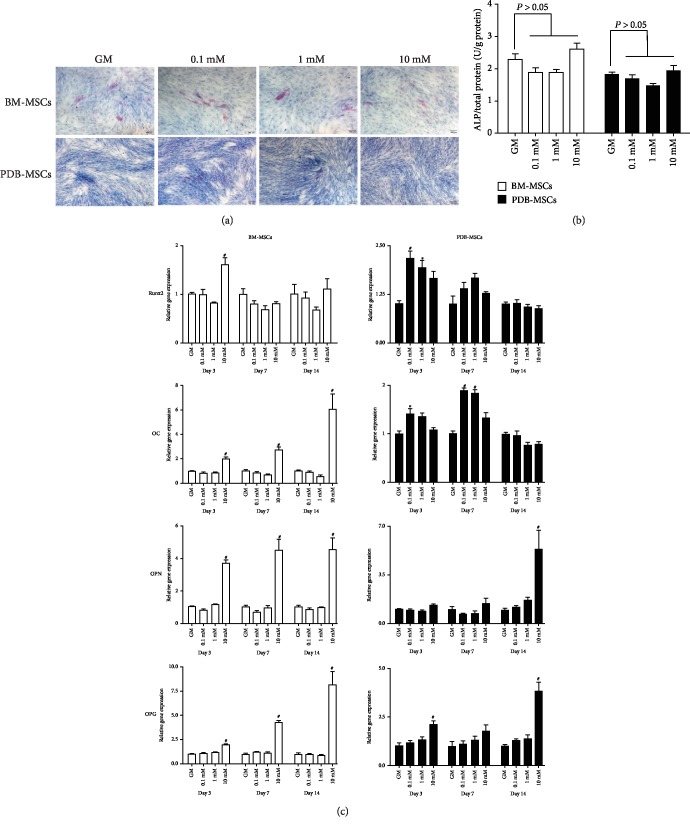
Effect of strontium on the osteogenic differentiation of BM- and PDB-MSCs cultured in the growth medium. (a) Results of the ALP staining of BM- and PDB-MSCs cultured with strontium for 7 days. Red indicates positive staining. Scale bar: 100 *μ*m. (b) The ALP activity of BM- and PDB-MSCs treated with strontium for 7 days, *n* = 4 for each group. (c) The osteogenic gene expression of BM- and PDB-MSCs on days 3, 7, and 14, *n* = 4 for each group. All of these results are representative of three independent experiments. ^∗^*P* < 0.05 when compared with the GM group; ^#^*P* < 0.01 when compared with the GM group. GM: growth medium.

As shown in Figure 4(a), only a few cells were positive for the ALP staining in both cell types after 7 days of culture, and there was no obvious difference among all groups in each cell type. Accordingly, the ALP activity of strontium groups (0.1 mM, 1 mM, and 10 mM) was comparable to that of the GM group (*P* > 0.05, Figure 4(b)). However, when comparing with the ALP staining of BM-MSCs, a much weaker staining result was found after PDB-MSCs were treated with the same concentration of strontium (Figure 4(a)).

The osteogenic gene expression of cells on days 3, 7, and 14, including the expression of *Runx2*, *OC*, *OPN*, and *OPG*, is shown in Figure 4(c). For BM-MSCs, 0.1 mM and 1 mM strontium demonstrated a similar gene expression level when compared with the GM group, while 10 mM strontium significantly enhanced the expression of *OC*, *OPN*, and *OPG* on days 3, 7, and 14, and the expression of *Runx2* on day 3. For PDB-MSCs, 0.1 mM and 1 mM strontium elevated the expression of *Runx2* on day 3, as well as the expression of *OC* on day 7. Furthermore, 10 mM strontium enhanced the expression of *OPN* and *OPG* on day 14.

Altogether, these results revealed that, even without any other soluble osteogenic factors, strontium promoted the osteogenic differentiation of both cells in a dose-dependent manner.

### 3.3. Strontium Promoted the Osteogenic Differentiation of BM- and PDB-MSCs in the Osteogenic Medium

We next determined whether strontium enhanced the osteogenic differentiation of BM- and PDB-MSCs in an osteogenic microenvironment *in vitro*. Both cells were cultured in the osteogenic medium supplemented with different doses of strontium. The osteogenic differentiation of cells was measured by the ALP activity on day 7, the expression of osteogenic genes on days 3, 7, and 14, and finally the mineralization of extracellular matrix on day 21.

**Figure 5:**
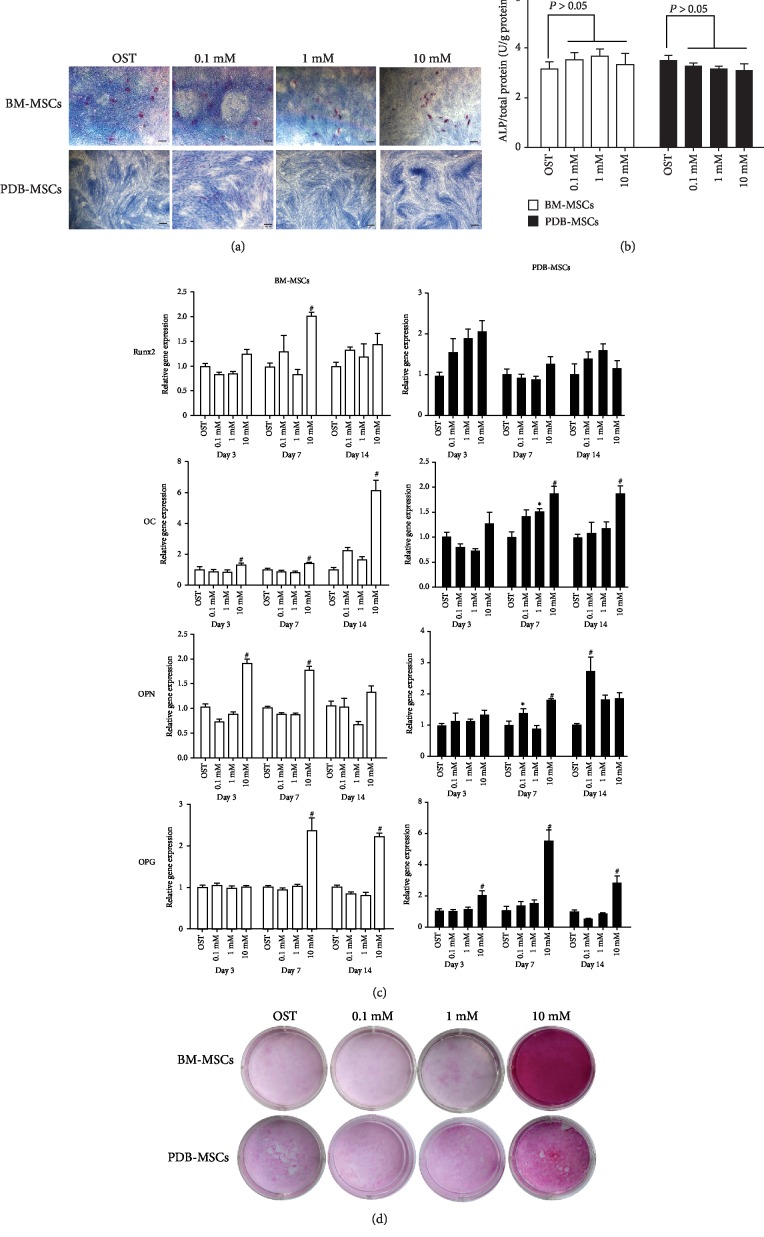
Effect of strontium on the osteogenic differentiation of BM- and PDB-MSCs cultured in the osteogenic medium. (a) ALP staining of BM- and PDB-MSCs cultured with strontium for 7 days. Red indicates positive staining. Scale bar: 100 *μ*m. (b) ALP activity of BM- and PDB-MSCs treated with strontium for 7 days. *n* = 4 for each group. (c) Osteogenic gene expression of BM- and PDB-MSCs on days 3, 7, and 14. *n* = 4 for each group. (d) Alizarin red staining of BM- and PDB-MSCs after 21 days of culture. Red indicates the mineralization of the extracellular matrix. All of these results are representative of three independent experiments. ^∗^*P* < 0.05 when compared with the OST group; ^#^*P* < 0.01 when compared with the OST group. OST: osteogenic medium.

As shown in Figure 5(a), both cells were positive for ALP staining after osteogenic induction, but there was no obvious difference among all groups in each cell type. Similarly, ALP activity of the strontium groups (0.1 mM, 1 mM, and 10 mM) was comparable to that of the osteogenic medium (OST) group on day 7 (*P* > 0.05, Figure 5(b)). Interestingly, BM-MSCs showed stronger staining results when comparing with that of PDB-MSCs after treated with the same concentration of strontium (Figure 5(a)).

As shown in Figure 5(c), strontium promoted the osteogenic gene expression of both cell types in a dose-dependent manner. For BM-MSCs, 0.1 mM and 1 mM strontium showed a comparable osteogenic gene expression level to that of the OST group, while 10 mM strontium enhanced the expression of *Runx2* on day 7, the expression of *OC* on days 3, 7, and 14, the expression of *OPN* on days 3 and 7, and the expression of *OPG* on days 7 and 14.

For PDB-MSCs, 0.1 mM strontium elevated the expression of *OPN* on days 7 and 14; 1 mM strontium enhanced the expression of *OC* on day 7; and notably, 10 mM strontium enhanced more osteogenic gene expressions than the 0.1 mM and 1 mM groups, facilitating the expression of *OC* on days 7 and 14, the expression of *OPN* on day 7, and the expression of *OPG* on days 3, 7, and 14 (Figure 5(c)).

The mineralization of cells was observed by the Alizarin red staining (Figure 5(d)). Compared with the OST group, 0.1 mM and 1 mM strontium showed a similar mineralization in both cell types, while 10 mM strontium obviously enhanced their mineralization.

Taken together, the above results clearly demonstrated that strontium can effectively promote the osteogenic differentiation of both BM- and PDB-MSCs under traditional osteogenic induction *in vitro*.

## 4. Discussion

PDB-MSCs are multipotent and readily available and, thus, present a potential cell source for bone repair. Nevertheless, the osteogenic potential of MSCs derived from the placenta tissues has been shown to be inferior to that of BM-MSCs [16–18], suggesting a need for effective osteogenic induction. As a trace element in natural human bone tissue, strontium pronouncedly enhances bone regeneration, mainly through stimulating the osteogenic differentiation of BM-MSCs while inhibiting the differentiation of osteoclasts [19]. In this study, we found that strontium facilitated the proliferation and osteogenic differentiation of PDB-MSCs, which shared a great similarity to the response of BM-MSCs. As far as we know, this is the first study to investigate the effect of strontium on the proliferation and osteogenic differentiation of PDB-MSCs *in vitro*.

According to our results, strontium exerted a dose-dependent effect on the proliferation of human PDB-MSCs, which was also observed in human BM-MSCs. Similarly, in other MSC cultures, such as rat BM-MSCs [26] and human adipose tissue-derived MSCs [37], strontium also exerted a dose-dependent effect on their proliferation. In this study, we found that 20 mM strontium inhibited the replication of both PDB- and BM-MSCs, while 1 mM, 5 mM, and 10 mM strontium promoted their proliferation. In the literature, different results have been reported regarding the cytotoxic effect of strontium on human BM-MSCs [29–31] and osteoblasts [38, 39]. For instance, it was found that strontium at concentrations of 1 mM and above drastically decreased the viability of human BM-MSCs [30]. However, 1 mM strontium was also reported to enhance the proliferation of human BM-MSCs by another research group [31]; additionally, it was determined that 210.7 *μ*g/mL strontium (i.e., 2.4 mM) did not inhibit the proliferation of human BM-MSCs [29]. These discrepancies associated with strontium cytotoxicity may be partially due to the following reasons: First, human BM-MSCs derived from different donors respond differently to strontium stimulation [31]. Second, the experiment conditions, including cell culture medium, cell passage, and methods used to test cytotoxicity, varied greatly among studies [29–31]. Considering this, it is difficult to compare them directly; instead, the discrepancies described above highlight the need to determine proper concentrations for experiment cells.

Unlike a proproliferation effect of the high concentrations of strontium (1 mM, 5 mM, and 10 mM) on both BM- and PDB-MSCs, they responded differently to some lower concentrations of strontium. For instance, 0.01 mM and 0.5 mM strontium increased the growth kinetics of human BM-MSCs but not that of PDB-MSCs. This cell-type-specific effect of strontium on the replication of somatic cells was also observed by other researchers [40], yet the mechanism underlying different cell responses remains largely unknown and needs further studies.

Regarding the osteogenic effects of strontium on MSCs, it has been reported that proper concentrations of strontium facilitated the osteogenic differentiation of rodent BM-MSCs [26–28], as well as MSCs derived from human tissues, such as bone marrow [29–31], umbilical cord [32], and adipose tissue [41]. In this study, we investigated, for the first time, the osteogenic effects of strontium on human PDB-MSCs, and found that 10 mM strontium, but not 0.1 mM or 1 mM strontium, promoted their osteogenic differentiation, which will provide a simple approach to stimulate the osteogenic potential of PDB-MSCs for bone regeneration.

Nevertheless, we should acknowledge that there are some limitations in this work. First, a pool of MSCs derived from four donors, but not cell replicates from each donor, was used in the experiments. Although this makes the comparison of MSCs derived from two different tissues simple and straightforward, it fails to uncover the donor-dependent variation in the responses of each cell type, which is important for future clinical applications. Second, the cells used in this study were at the middle passages. It is well-known that MSC-based therapies need a large number of cells, and that MSCs at passage 3-7 are usually used as graft cells in clinics [42]; therefore, we used both cell types at passage 5-7 in this study, which were relevant to the clinics. However, it should be noted that the differentiation potential of human MSCs declines with increasing passage number [43], and consequently, further studies are suggested to determine the proper cell passage for strontium stimulation. Last but not least, the mechanism underlining the osteogenic effects of strontium on human PDB-MSCs remains unknown. It has been reported that strontium enhanced the osteogenic differentiation of MSCs through different cell signaling pathways, such as the Ras/MAPK pathway [28], the Wnt/*β*-catenin pathway [32], and the MAPK/ERK pathway [44]. However, it is still unknown whether these signaling pathways are involved in the osteogenic differentiation of PDB-MSCs treated with strontium, and thus further studies are required to understand the target pathway.

Based on the above studies, it is clearly demonstrated that 10 mM strontium, rather than 0.1 mM and 1 mM strontium, obviously facilitated the proliferation and osteogenic lineage commitment of human PDB- and BM-MSCs. Considering the dose-dependent effect of strontium on the proliferation and differentiation of human MSCs, it is noteworthy to determine the proper ion release profile of strontium-containing bone scaffolds when using these MSCs to fabricate tissue engineered bone grafts.

## 5. Conclusions

In conclusion, strontium promoted the proliferation and osteogenic differentiation of human PDB- and BM-MSCs in a dose-dependent manner. At low doses of strontium (0.1 mM and 1 mM), the osteogenic gene expression of PDB-MSCs was slightly different from that of BM-MSCs, but the mineralization of both cell types was not enhanced. By contrast, they shared a great similarity in the response to a relative high dose of strontium (10 mM), which promoted their proliferation and osteogenic differentiation. This dose-dependent effect of strontium should be taken into consideration when combining strontium and human MSCs for bone regeneration.

## Data Availability

The data used to support the findings of this study are included within the article.

## Abbreviations

ALP: Alkaline phosphatase
BM-MSCs: Bone marrow-derived mesenchymal stromal cells
*GADPH*: Glyceraldehyde-3-phosphate dehydrogenase
GM: Growth medium
*OC*: Osteocalcin
OST: Osteogenic medium
*OPG*: Osteoprotegerin
*OPN*: Osteopontin
PDB-MSCs: Placental decidual basalis-derived mesenchymal stromal cells
*Runx2*: Runt-related transcription factor 2.
